# Using behaviour change and implementation science to address low referral rates in oncology

**DOI:** 10.1186/s12913-018-3653-1

**Published:** 2018-11-28

**Authors:** Janet C. Long, Deborah Debono, Rachel Williams, Elizabeth Salisbury, Sharron O’Neill, Elizabeth Eykman, Jordan Butler, Robert Rawson, Kim-Chi Phan-Thien, Stephen R. Thompson, Jeffrey Braithwaite, Melvin Chin, Natalie Taylor

**Affiliations:** 1 Centre for Healthcare Resilience and Implementation Science, Australian Institute of Health Innovation, Faculty of Medicine and Health, Macquarie University, Sydney, NSW Australia; 2 Faculty of Health, University of Technology, Sydney, Australia; 3 Nelune Comprehensive Cancer Centre, Prince of Wales Hospital, Randwick, NSW Australia; 4 Prince of Wales Clinical School, Faculty of Medicine, University of New South Wales, Sydney, NSW Australia; 5 NSW Pathology (SEALS), Prince of Wales Hospital, Randwick, NSW Australia; 6 School of Business, University of NSW, Campbell, ACT Australia; 7 NSW Pathology (SEALS), St George Hospital, Kogarah, NSW Australia; 8 Anatomical Pathology, Royal Prince Alfred Hospital, Camperdown, NSW Australia; 9 St George and Sutherland Clinical School, University of New South Wales, Randwick, NSW Australia; 10 Cancer Council NSW, Woolloomooloo, NSW Australia

**Keywords:** Implementation, Theoretical domains framework, Behaviour change, Hereditary cancer, Pathology, Referral, Systems change

## Abstract

**Background:**

Patients undergoing surgery for bowel cancer now have a routine screening test to assess their genetic predisposition to this and other cancers (Lynch syndrome). A result indicating a high risk should trigger referral to a genetic clinic for diagnostic testing, information, and management. Appropriate management of Lynch syndrome lowers morbidity and mortality from cancer for patients and their family, but referral rates are low. The aim of this project was to increase referral rates for patients at high risk of Lynch syndrome at two Australian hospitals, using the Theoretical Domains Framework (TDF) Implementation approach.

**Methods:**

Multidisciplinary teams at each hospital mapped the referral process and discussed barriers to referral. A 12-month retrospective audit measured baseline referral rates. The validated *Influences on Patient Safety Behaviours Questionnaire* was administered to evaluate barriers using the TDF. Results were discussed in focus groups and interviews, and interventions co-designed, guided by theory. Continuous monitoring audits assessed change in referral rates.

**Results:**

Teams (*n* = 8, 11) at each hospital mapped referral processes. Baseline referral rates were 80% (4/5) from 71 screened patients and 8% (1/14) from 113 patients respectively. The questionnaire response rate was 51% (36/71). Most significant barrier domains were: ‘environmental context;’ ‘memory and decision making;’ ‘skills;’ and ‘beliefs about capabilities.’ Focus groups and interviews with 19 healthcare professionals confirmed these domains as significant. Fifteen interventions were proposed considering both emerging and theory-based results. Interventions included: clarification of pathology reports, education, introduction of e-referrals, and inclusion of genetic status in documentation.

Audits continued to December 2016 showing a change in pathology processes which increased the accuracy of screening. The referral rate remained low: 46% at Hospital A and 9% Hospital B. Results suggest patients who have their referral deferred for some reason are not referred later.

**Conclusion:**

Lynch syndrome is typical of low incidence problems likely to overwhelm the system as genomic testing becomes mainstream. It is crucial for health researchers to test methods and define generalizable solutions to address this problem. Whilst our approach did not improve referrals, we have deepened our understanding of barriers to referral and approaches to low frequency conditions.

**Electronic supplementary material:**

The online version of this article (10.1186/s12913-018-3653-1) contains supplementary material, which is available to authorized users.

## Background

A key challenge to the delivery of safe, high quality health services is healthcare professionals’ ability to remain abreast of new research and to translate these new understandings into feasible and appropriate clinical practice [1]. One field in particular that is rapidly generating new clinically relevant research and guidelines which require an active implementation effort is cancer genomics. Whilst there is incredible potential for these scientific discoveries to improve diagnosis and treatment of patients, the translation of each new piece of genetic evidence into clinical practice is complex, challenging, and slow [2].

One such example is Lynch Syndrome (LS). LS is a hereditary condition associated with a high incidence of colorectal, endometrial and a range of other cancers [3, 4]. Screening tests that identify people with a high risk of LS have been shown to reduce morbidity and mortality and to be cost effective [5, 6]. Screening consists of consideration of the patient’s clinical presentation, family history and pathology testing of a tumour sample. Universal screening is not yet fully implemented in Australian hospitals with individual pathology and oncology departments applying an ad hoc screening practice. Currently, all patients undergoing surgery for CRC have routine immunohistochemistry (IHC) for four mismatch repair genes performed on their tumour to determine risk of LS. A secondary screening test (IHC for BRAF V600E) for a subset of patients may now also be performed to distinguish tumours likely to be due to a faulty gene from those that are not. At the commencement of the study, this secondary screening test was not in place at either pathology department. However, one of the pathology laboratories commenced this more than half way through the study. Patients with CRC who present with additional clinical factors such as a young age or a strong family history of bowel cancer may be flagged as having a high risk of LS before surgery, but the IHC is still performed to help identify which of the four Mismatch Repair genes are involved. High risk results are ideally acted on by referring the patient to a Familial Cancer Clinic (FCC), which provides counselling, assesses the risk further and arranges diagnostic genetic testing if appropriate [7]. Once diagnosed, risk can be managed by appropriate surveillance, and consideration of prophylactic surgery. Family members can also be informed and tested, as appropriate. As an autosomal dominant condition, family members have a 50% chance of inheriting the faulty gene. Lynch Syndrome Australia, an advocacy group for people living with LS, stresses the importance of access to high quality information and counselling from a genetic service, ideally an FCC as part of the testing process [8]. Australian [6, 9, 10] and international [11–13] evidence however, indicates that LS is underdiagnosed, one cause of which is low referral to genetic services of at risk patients identified by screening tests. This paper presents the case of LS, where local concerns of genetic specialists, and (unpublished) audits of screening tests being incorporated into practice over the last five years in Australian hospitals, have not yet succeeded in raising the low rate of diagnosis of this hereditary cancer syndrome.

Implementation science recognises that practice change is often not achieved simply by disseminating the findings of the latest clinical trial or systematic review [14]. An understanding of context, current work flows, and barriers to change are essential to tailor new interventions to be an acceptable fit with existing practices. At the same time, there is a need for effective, generalizable, and replicable interventions suitable for similar healthcare contexts to avoid unnecessary replication and effort [15]. An understanding of the theoretical underpinnings of both the problem (barriers) and the use of evidence based and pragmatic solutions (interventions) can greatly assist this. Here we demonstrate how we used the validated, six step Theoretical Domains Framework Implementation (TDFI) approach – which combines behaviour change theory with implementation science, to translate LS evidence into practice [16–18].

The aim of our project was to increase recognition of high risk patients and subsequent referrals to the FCC. We used the TDFI approach to structure activities to address the problem [17], using audits of IHC results and referrals to assess change.

## Methods

### Overview

**Fig. 1:**
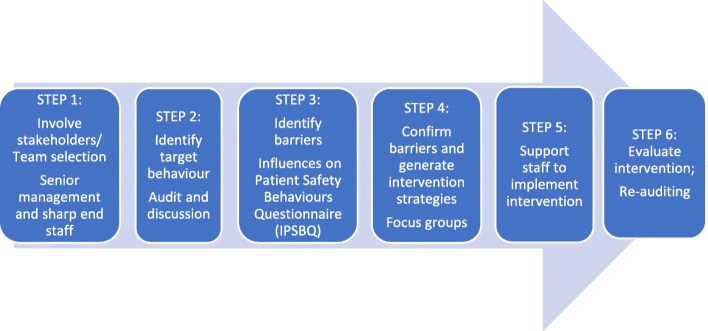
The Theoretical Domains Framework Implementation Approach [19]

The study was conducted in two large Australian hospitals with no clearly documented screening protocol but wide circulation of Evi-Q guidelines [7] (evidence-based guidelines used in Australia to guide cancer treatment and management). There was evidence clinicians’ familiarity with clinical presentations and family history that suggested a high risk of LS. Hospital A performed surgery on around 75 patients with CRC per year, while Hospital B performed surgery on around 120 patients with CRC per year. Investigators were drawn from health services research, clinical practice (genetics, surgery, medical and radiation oncology and pathology) and two experienced consumer partners, one living with LS. The six step TDFI process was as follows. Step 1: multidisciplinary implementation teams were formed at each hospital to process map LS referrals and discuss barriers. Step 2: a baseline audit of CRC surgery patients and LS genetic testing referrals were completed at each hospital to populate the process maps with objective data and determine the extent of the problem. Step 3: health professionals involved with CRC patients were invited to complete the validated *Influences on Patient Safety Behaviours Questionnaire* (*IPSBQ*) [19] to identify barriers to referrals, which was followed up by TDF-guided focus groups to verify the referral barriers. Step 4: interventions were co-designed with health professionals using evidence-based behaviour change techniques (BCTs) to address key barriers. Step 5: interventions were implemented. Step 6: the effectiveness of the project was evaluated using ongoing audit (see Fig. 1). Ethical approval and site specific governance was granted for this study by the local health district’s Human Research Ethics Committee (reference: 15/103).

### Step 1: Multidisciplinary implementation team formation

Multidisciplinary implementation teams at the two hospitals were recruited through targeted invitation of known key staff and via expressions of interest. Teams were made up of people involved in the referral process and included medical and radiation oncologists, colorectal surgeons, anatomical pathologists, and genetic counsellors. Each team mapped the process of referral in their hospitals based on the typical patient journey. Process maps were sketched out on paper or by taking detailed notes with project investigators, then created electronically using Microsoft Visio software by the health services researchers. A narrative outline of the steps in the referral process was developed using exploratory questions (e.g., “and then what happens?”) and refined and clarified over subsequent iterations. Seven face-to-face sessions were conducted between April and August 2015. Health services researchers facilitated all meetings and kept a project log to document project teams’ assumptions and subjective views of the referral process, as well as discussions around barriers to referral and suggested solutions.

### Step 2 audits and defining the target behaviour

The process maps defined key points where audit data could be collected, to both test assumptions of compliance with various steps, and to highlight problem areas on which to focus effort (e.g., number of people identified as having a high risk of LS who were not referred, or referred but chose not to attend FCC). Audits were timed to assess baseline, project initiation and intervention stages of the project for changes in referral numbers and processes. Data collected at each hospital were: patient’s age, date of surgery, date of specimen receipt at pathology, date of report authorisation, date of supplementary report authorisation (if applicable), IHC results, BRAF V600E requests/results (if applicable), comments made by the reporting pathologist, presence and date of referral to FCC, acceptance by patient of referral, and attendance of the patient at the FCC. Referrals made to public genetic clinics elsewhere in the state were also noted. No clinical or family history was collected.

Audit data was collected by pathologists in the two hospitals searching their report databases using Systematised Nomenclature of Medicine (SNOMED) clinical terms to find reports on specimens coded as large, complex colorectal tumour specimens. IHC results and timing of reports were extracted from the retrieved reports. A genetic counsellor then matched patients to FCC clinic records by searching the statewide genetic service database. Records were then de-identified so whole numbers could be collated. Baseline data was collected retrospectively and covered the period May 2014–April 2015. The project initiation phase covered the months during which the project and the problem were discussed but no formal interventions had been started (May 2015–January 2016). The intervention phase covered the period February to November 2016. Numbers of patients flagged as at high risk were as per EviQ guidelines [20], and the number of those that were referred were graphed on a quarterly run chart.

The TDFI approach requires the team to define a target behaviour; that is, a behaviour that, if performed optimally, would result in the desired practice change. Results from the process mapping activities and the audit results were considered by an expert panel (oncologist, genetic counsellor and health services researchers) in order to define the target behaviour.

### Step 3 influences on patient safety Behaviours questionnaire (IPSBQ)

The *IPSBQ* was used to determine barriers to achieving the target behaviour (“ensuring patients identified as having a high risk of Lynch syndrome are referred to genetic counselling”) by considering each of 11 Theoretical Domain Framework (TDF) barriers (e.g. emotion, beliefs about consequences) [21]. The expert panel that defined the target behaviour also designed and formatted the *IPSBQ* to our context and purpose. The five minute *IPSBQ* was offered in paper and online format to all staff involved in the management of patients with CRC. The top four barriers were determined by ranking the average scores for each domain (positively worded items were reverse-scored); the highest ranked scores represented the most significant barriers). We used the *IPSBQ* Data Entry Spreadsheet to compute these values [19]. ANOVAs were used to determine intergroup differences for Hospital A/Hospital B; those who refer/those that do not; those who were familiar with the guidelines for referral/those who were not. Significance was set at α < 0.05. Barrier domains were tested for internal reliability across questions using Pearson correlations (one-tailed) for domains with two items (with values between *r* = 0.15–0.50 being acceptable correlation [22, 23]) and Cronbach’s alpha for the barrier with three items (with α > 0.70 being considered acceptable [24]).

### Step 4 focus groups and co-design of interventions

Focus group participants were recruited using a purposive sampling approach to ensure a wide range of experience and viewpoints from staff involved in the management of people with CRC. We recruited via email and Colorectal Cancer Multi-Disciplinary Team meetings and offered separate interviews for people unable to schedule attendance at a group. Both types of session were digitally recorded or hand scribed and transcribed. The format of focus groups and interviews were the same, following a schedule of resources and questions (Additional file 1). This described the domains used to categorise the barriers, BCTs that could address each barrier domain, and examples of strategies identified in the literature that used appropriately matched BCTs e.g. [19, 25, 26].s For example, ‘adding objects to the environment’, identified as an effective BCT to address barriers related to the ‘environmental context and resources’ domain, has been previously operationalised by ensuring availability of pH paper to improve compliance with testing of aspirate as the first line check for correct positioning of nasogastric tubes [19]. Interventions discussed reflected the different roles of participants: those who were responsible for the decision to refer (treating oncologists and surgeons) and those in a supportive role (e.g. pathologists, geneticists, nurses).

In an iterative, bi-directional process, a table was constructed to clarify theoretical relationships [27–29]. Interventions generated by the focus groups and interviews were entered into this table alongside associated barrier domains. Log data of interventions that had been intuitively suggested were also added to the table and categorised according to the TDF domain being addressed. Results were triangulated with the *IPSBQ* data, and suggested interventions that did not address one of the four highest scoring barriers were removed. Final intervention strategies used to address key barriers emerged from synthesis of the intuitive and theory-based methods. Expert knowledge within the group (NT, DD, JL) was used to match the appropriate BCT from Abraham’s taxonomy [30] to each barrier.

### Step 5 implementation of the interventions

Final versions of the proposed interventions were provided to hospital and department directors, and their authorisation and support for their implementation was formally sought. Health services researchers facilitated intervention development wherever possible.

### Step 6 monitoring of results

Intervention phase audit results were compared to baseline and project initiation phase results. The interventions started in 2016 were assessed for level of completion at the end of the year and were treated as an intervention bundle when assessing their impact and outcomes.

## Results

### Step 1 implementation team selection and process mapping

Key health professionals at each oncology department were recruited to the multidisciplinary implementation teams: Hospital A (*n* = 8) and Hospital B (*n* = 11). The teams mapped the process of referral based on the patient journey. There were nine iterations of the map. The maps showed that IHC testing was done on all tumours removed from CRC patients (initiated by pathologists, but not an enforced policy) and that referral of high risk patients was reliant on correct interpretation of the ensuing report, and faxing of a paper referral form or letter to the FCC. Nine suggestions of interventions (intuitive interventions) were recorded in the project Log.

### Step 2 audits

**Fig. 2:**
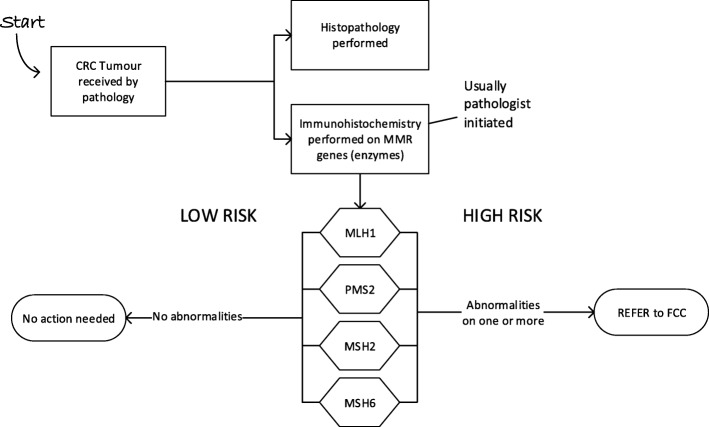
Pathology flowchart at start of the project. All abnormalities found were referred directly to the Familial Cancer Clinic (FCC)

The 12-month retrospective audit carried out by pathology registrars at each hospital found 71 and 113 patients having surgery for CRC at Hospital A and Hospital B respectively. Criteria for referral (following EviQ guidelines [20]) was ‘any patient, of any age that had an abnormality on screening on one or more of the four mismatch repair genes (MLH1, PMS2, MSH2, MSH6)’ (see Fig. 2). Supplementary testing (BRAF V600E) was not available at this time at either hospital pathology departments, so patients with MLH1/PMS2 abnormalities could only be referred to FCC, who then arranged supplementary testing through another pathology service. Hospital A had 11 patients with high risk results and referred four (36%) and Hospital B, had 16 patients and referred two (13%), showing that referral rates were suboptimal. Of the high risk patients who were not referred during this period, all were 60 years or older.

Baseline audit data from each hospital were added to simplified process maps, matched to the appropriate step. These maps were used as the main communication and discussion tool, and alongside the audit were used to establish the target behaviour, which was defined as: “ensuring that every patient at high risk of Lynch syndrome is referred to genetic counselling.” The generic nature of the target behaviour reflected the differing roles and responsibilities of the staff involved in management of patients with CRC. This phrase completed the 23 barriers statements in the *IPSBQ* (see Table 1). Respondents indicated agreement or disagreement with the statements on a five point Likert scale (1 = strongly agree to 5 = strongly disagree).

**Table 1:** Statements in the *Influences on Patient Safety Behaviours Questionnaire* matched to the 11 domains of barriers to behaviour change [21]

Domain	Questions	Target behaviour
Knowledge	I know what the guidelines say about the need to … I fully agree with the guidelines which instruct staff to …	*… ensure that every patient at high risk of Lynch syndrome is referred to genetic counselling.*
Skills	*Training is not offered to me regularly enough to … *Training is not adequate to …	
Social/profession role and identity	*It isn’t my responsibility to … I am clear about what my role should be in the process to …	
Beliefs about capabilities	*I do not find it easy to … *I have previously encountered problems when trying to …	
Beliefs about consequences	*It does not matter too much if I do not… It will be bad for the patient if I do not …	
Motivation and goals	*Emergencies and other priorities get in the way of me being able to … *Other guidelines conflict with me being able to …	
Memory, attention and decision-making processes	I habitually (or usually) … *There are justifiable reasons why often decide not to …	
Environmental context and resources	*There is not a good enough system in place to … I have the necessary resources (e.g. correct/enough equipment, staff, etc) to do … Verbal and written communication between staff is clear enough for me to…	
Social influences	*Other staff don’t seem to … My superiors would like me to …	
Emotion	*I feel anxious if I think about having to … *I worry if I think about having to …	
Behavioural regulation/action planning	*Plans in my head often get muddled when trying to … *Things are too unpredictable to make plans to …	

Respondents indicated agreement or disagreement with the statements on a five point Likert scale: *(1 = strongly agree to 5 = strongly disagree)*

Note: Each question is ended by the stated target behaviour: “*to ensure that every patient at high risk of Lynch syndrome is referred to genetic counselling.”* Questions marked with a *are stated as barriers and scores were reversed for analysis

### Step 3 ISPBQ

The three-minute *IPSBQ* was formatted to include the target behaviour and circulated to 71 healthcare professionals with a role in treatment or management of patients with CRC. The response rate was 51% (36/71). The majority of respondents (24/36) were medical officers with the rest from nursing (6), genetics (4) and administration (2). Sixteen (44%) respondents indicated that they had responsibility for making referrals. Guidelines were familiar to 69% (25/36). Table 2 outlines respondents’ details.

**Table 2:** Details of the *Influences on Patient Safety Barriers Questionnaire* respondents

Specialty or work area	n	%
Medical oncology^a^	5	14
Surgery^a^	8	22
Pathology	5	14
Familial Cancer	6	17
Genetics Service admin	1	3
Radiation oncology^a^	3	8
Oncology nursing	6	17
Oncology admin	1	3
Palliative care	1	3
Years of experience	n	%
0–1	5	14
2–5	14	39
Over 5	17	47
Responsible to refer	n	%
Yes	16	44
No	20	56
Familiarity with Guidelines	n	%
Yes	25	69
No	11	31

^a^denotes groups on the treating team with individual or joint responsibility to refer

Missing values analysis (MVA) was undertaken on the full data set (*n* = 36) to highlight patterns of missing data and to replace them [31]. Little’s MCAR test [32] was not significant (*p* = 0.23) indicating that data was missing completely at random. Therefore, estimation maximisation (EM) was used to impute missing values.

The highest scoring domain for barriers was ‘environmental context and resources’ [mean (M) 3.08, standard deviation (SD) 0.84], followed by ‘skills’ (M = 2.78, SD = 1.17), ‘beliefs about capabilities’ (M = 2.75, SD = 1.16), ‘social influences’ (M = 2.73, SD = 0.76), and ‘memory, attention and decision making’ (M = 2.61, SD = 0.84) (see Table 3).

**Table 3:** Top ranked barrier domains for the different groups of respondents

Barrier Domain	Mean score	Standard deviation
All respondents *n* = 36		
Environmental context and resources	3.08	0.84
Skills	2.78	1.17
Beliefs about capabilities	2.75	1.16
Memory, attention and decision-making	2.61	0.84
Responsible for referral *n* = 16		
Environmental context and resources	3.00	0.88
Beliefs about capabilities	2.76	1.19
Memory, attention and decision-making	2.50	0.80
Skills	2.24	0.90
Not responsible to refer *n* = 20		
Skills	3.16	1.19
Environmental context and resources	3.12	0.79
Memory, attention and decision-making	2.69	0.89
Beliefs about capabilities	2.64	1.04
Familiar with referral guidelines *n* = 25		
Environmental context and resources	3.06	0.86
Beliefs about capabilities	2.63	1.20
Memory, attention and decision-making	2.57	0.94
Skills	2.55	1.07
Not familiar with referral guidelines *n* = 11		
Skills	3.22	1.24
Environmental context and resources	3.09	0.75
Beliefs about capabilities	2.84	0.82
Professional identity	2.74	0.72
Memory, attention and decision-making	2.68	0.60

Internal reliability was demonstrated by significant inter-item correlations and Cronbach’s alpha for nine of the eleven domains; ‘social/professional identity,’ and ‘social influences’ domains did not demonstrate internal reliability and were removed from the analysis (See Table 4).

**Table 4:** Inter-item reliability scores for barriers

Domain	Number of questions	Pearson’s correlation	Significance (one-tailed)	Cronbach’s Alpha
Knowledge	2	**0.44**	**0.00**	
Skills	2	**0.61**	**0.00**	
Social/profession role and identity	2	0.01	0.28	
Beliefs about capabilities	2	**0.36**	**0.02**	
Beliefs about consequences	2	**0.39**	**0.01**	
Motivation and goals	2	**0.44**	**0.01**	
Memory, attention and decision-making	2	**0.34**	**0.02**	
Environmental context and resources	3			**0.70**
Social influences	2	0.07	0.35	
Emotion	2	**0.64**	**0.00**	
Behavioural regulation/action planning	2	**0.56**	**0.00**	

One-way ANOVAs with Hospital, and then responsibility to refer as the grouping variable, showed no significant difference in any of the barriers. Additional file 2 shows details.

### Step 4

Focus groups and individual consultations involving nineteen key healthcare professionals confirmed that these five domains rang true and that ‘environmental context and resources’ represented the most significant barrier. Fifteen interventions were proposed by considering both emerging and theory-based results. Executive support was given for 12 of these interventions.

### Step 5

Interventions were co-designed using appropriate BCTs matched to the identified barrier domains. For example, the ‘memory, attention and decision making’ domain may be addressed by the BCT of using ‘prompts and cues’ to focus attention, aid in decision making and streamline workflow. One team decided to add a pro forma at key follow-up consultations to review the patient’s risk of hereditary cancer and to check if a referral had been made. This was a clear prompt to the team, making referral less likely to be overlooked. Table 5 lists details of barrier domains, barriers, proposed intuitive and theory based interventions, and matched BCTs. Also included are notes on how we operationalised the interventions.

**Table 5:** Details of barrier domains, barriers, proposed intuitive interventions, proposed theory-based interventions, and matched behaviour change techniques

Barrier Domain	Description of actual barrier	Proposed intuitive interventions	Proposed theory-based interventions	Details of final Intervention Strategy (Progress: Not adopted (NA); Not yet started (NS); In progress (P); Completed (C); Ongoing (O)	Behaviour Change Technique (BCT)
Environmental context and resources *Staff perceptions include: the system is not good enough/the necessary resources/verbal and written communication are not adequate to ensure high risk patients are referred.*	Paper FCC referral forms not always available in clinic; faxing process can be fraught; multiple electronic management systems being used across hospitals and departments with limited connectivity.	Concerted effort be made to restock paper referral forms in the clinics [Log] Write a letter as a referral to overcome problem of unavailable forms [Log] Put the referral forms on Hospital B’s electronic patient management system [Log]	Put the referral forms on both Hospitals’ respective electronic patient management systems [Focus groups] Enable emailed referrals for those clinicians who have the form template but do not have access to the hospital’s electronic patient management system. Forms are currently faxed. [Focus groups]	Genetic staff and data managers to put referral forms on each hospital’s electronic patient management system and optimise known limited interoperability with FCC database. (P) Add an email address to paper or printable versions of forms. Replace printable form on hospital forms site and email to private referrers with explanation of changes. (C) Genetic staff to develop protocol for retrieving referrals, adding patient details to genetic database (different from the oncology database), triaging and booking appointments. (C) Genetic staff and referring clinicians to develop documenting regimes to: allow treating team to track referral progress, and to record if patients decline or defer an appointment. (P) Use colorectal group email, and oncology department and multidisciplinary team meetings to disseminate information about how to refer on each system and where to look for pending or completed appointments. (O)	Adding objects to the environment; Instruction on how to perform the behaviour Adding objects to the environment; Instruction on how to perform behaviour Self-monitoring of outcomes of behaviour Adding objects to the environment; Restructuring the physical environment; Social support (practical) Instruction on how to perform the behaviour; Credible source
	Referral forms are not seen as flexible enough	Forms were easy to fill out (check the box) but more room was needed to explain atypical presentations. [Interview]		Genetic staff and referring clinicians to review content of referral forms and include a larger free text box for referrals that do not meet the tick box criteria (C)	Restructuring the physical environment; Adding objects to the environment;
	Reflex secondary testing (BRAF V600E testing for MLH1 abnormal specimens) has been agreed in principle by pathology department but implementation of in-house testing is delayed		Of all the high risk IHC results, ones involving abnormal MLH1 were the most likely to not be followed up. [Audit]	Pathology department to develop a departmental protocol to automatically send MLH1 abnormal specimens to outside pathology services for BRAF V600E testing. (C) Until this happens, treating team to order BRAF V600E testing. Instructions and reminders to be given at CRC meetings by genetic and pathology staff. (C)	Adding objects to the environment; Conserve mental resources Instruction on how to perform the behaviour; Social support (practical); Cues and prompts
	Pathology and hereditary cancer representatives provide valuable expertise on appropriate referrals to multidisciplinary team meetings (case conferences) but are not always available for meeting at Hospital B. Patient information is not always made available before the meetings.		Change the time of the multidisciplinary meeting at Hospital B to a time when pathology and genetic service representatives could attend [Focus group]	Changing the time of the Multidisciplinary meeting was investigated but deemed not feasible. (NA) Multidisciplinary team coordinator to add a genetics field to the patient information template circulated before the meeting and remind clinicians presenting patients that each field needs to be discussed. If pathology and genetic representatives are unable to attend the meeting they can review this information that is circulated before the meeting and flag issues for the chairperson to raise (P)	n/a Social support (practical); cues and prompts
Skills and knowledge S*taff perceptions include: training is not adequate/ offered regularly enough; lack of awareness about/ agreement with/ understanding about/the guidelines*	Surgical teams may not be familiar with latest referral guidelines		Ensure surgeons are included in education around hereditary cancer referral criteria updates [Focus groups]	Presentation to Surgical Grand Rounds on best practice in referral for patients flagged as having a high risk of Lynch Syndrome. (C)	Credible source; Information about health consequences
	Rotating staff may not be familiar with the referral process. Currently training is ad hoc		A session on how to explain hereditary cancer risk to patients, how to refer, and how to interpret results to be offered to rotating staff [Focus group]	Quarterly workshops for new surgical and oncology Medical Officers on Hereditary Cancer including pathology and referral processes to be established. (O)	Instruction on how to perform the behaviour; Credible source; Information about health consequences
	Supplementary testing for patients with MLH1 abnormalities was not routinely being ordered by treating clinicians/not yet initiated by pathology meaning patients were missed		Feedback of audit results showing that these patients were the largest group not receiving appropriate action	Feedback of audit results to key treating clinicians with genetic and pathology specialists in attendance to highlight the number of patients missing appropriate supplementary testing. Explanation of how to order and interpret BRAF V600E testing. (C)	Feedback on outcome of behaviour; instruction on how to perform the behaviour; credible source
	Oncology nurses have a role to play in helping identify patients with high risk family history, but are unsure of criteria. This recognises that family history is often not disclosed to the admitting officer but emerges later as the patient discusses it with his or her family. Nurses are the usual people who are told this additional information.	The nurses in one area were trained to recognise and pass on new relevant information disclosed by the patient, to the medical team. This should be replicated in other areas [Log]	Oncology nurses and allied health (social worker/counsellor, physiotherapists, occupational therapists, etc) to be provided with a training session	In-service education (30 mins with Powerpoint slides and a summary handout) for nursing and allied health staff. Objectives: to provide information about Lynch syndrome and accompanying increased cancer risks, to clarify what family history is relevant and what not, and how to communicate this information to the treating team or genetic service advocate. (C)	Information about health consequences; Credible source;
Beliefs about capabilities *Staff do not find it easy/have previously encountered problems when trying to refer*	Terminology in the pathology reports can be confusing to clinicians, pathologists and geneticists, generating the perception that it is hard to make an appropriate referral. Currently a mix of terms used: “positive/negative,” “abnormal/normal,” “preserved/lost”	Wording on the reports to be simplified and standardised [Log]	Wording on the reports to be simplified and standardised following the Royal Australian College of Pathologists’ recommended wording [Log]	Wording for IHC and BRAF V600E pathology reporting to be simplified and standardised following the Royal Australian College of Pathologists’ recommended wording to make results easier to interpret. (C)	Instruction on how to perform the behaviour; Credible source; Conserve mental resources
Memory, attention and decision making processes *Staff perceptions include: not having referral as habitual practice/having justifiable reasons for not referring*	Interpreting pathology results can be difficult, making the decision-making process more difficult and less routine		Small posters giving information about how to interpret IHC results to be put up in the clinics where patients come for follow-up [Focus groups]	Information sheets on how to interpret and act on IHC /BRAF V600E results for the surgical and oncology clinics where patients come for follow-up. (NS) [Not supported at Hospital B]	Instruction on how to perform the behaviour; Credible source; Prompts or cues
	A number of factors mean that referrals may be overlooked: e.g. IHC reports not available at patients’ first follow-up, competing priorities in limited consult time, delay (with potential to not follow up) e.g., when clinical judgement says patient is overwhelmed, or seriously ill and is unable to discuss a genetic referral	Document in the patient’s notes when a referral is postponed so it can be addressed next consult [Log]	Use a template that includes a genetics field for when patients are presented at case conference or for letters to external health providers [Focus groups and interviews]	Incorporation of IHC results and genetic referrals (or pending referrals) to be included routinely in correspondence from multidisciplinary case conferences to patients’ external healthcare providers. (P) Use a checklist or documentation protocol for follow up consultations to ensure that when genetic referrals are not addressed post-operatively for whatever reason, they are not overlooked or forgotten entirely. Checklist being introduced by one team for follow up consults. (P)	Prompts or cues; Social support (practical); Credible source Prompts or cues; Social support (practical)

Unless specified, interventions were department wide, involving both hospitals’ oncology, FCC and/or pathology departments

Interventions were co-designed: clinicians brought experience, knowledge of existing work flow and tacit knowledge of processes to the table, while researchers advised on the best theory-based approaches and ways to study and map processes. Researchers also provided some practical support for interventions that were stalled or delayed due to lack of internal organisational capacity (e.g. by formatting new forms, liaising with data managers).

By mid-2016 there were enough results from ongoing audits to analyse trends in the referral patterns. An audit and feedback intervention was held with teams from each hospital at which a pathologist and a hereditary cancer specialist attended to answer questions. All patients under 50 years of age with abnormal IHC had been referred, often before their surgery, presumably on the basis of their family and clinical history. Patients over 50 years old with MLH1 and PMS2 abnormality were the main category not receiving referral.

### Step 6 monitoring of results

**Fig. 3:**
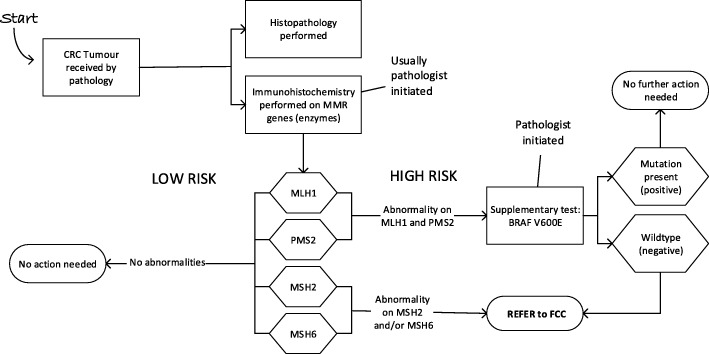
Pathology flowchart at end of the project. All patients with abnormalities on MSH2 and/or MSH6 were referred directly to the Familial Cancer Clinic (FCC), while patients with MLH1 and PMS2 abnormalities had a pathologist initiated supplementary test on the same tumour sample to determine the likelihood of a germline or somatic tumour

The audits continued to December 2016. Two things were revealed by the monitoring audits. First, a clear change in practice for patients over 50 years old with MLH1 and PMS2 abnormalities was now evident. Pathologists were consistently ordering supplementary testing for patients with MLH1 and PMS2 abnormalities (reflex testing). The results of this supplementary test (BRAF V600E) moves some patients from the high risk to the low risk group thus improving the ability to identify *appropriate* patients to refer. Responsibility for the initiation of this test had moved throughout the project from the FCC (at the start of the project), to the treating team (end 2015- mid 2016), to the pathologist (last quarter of 2016). The FCC could only initiate the test if a referral was first made. Figure 3 shows the new pathology process. As pathologists took over the role of initiating BRAF V600E testing, the proportion of appropriate patients recommended for this supplementary test went from 0% at Hospital A and 1% at Hospital B at baseline (2014) to 67 and 88% by mid-2016. In the final quarter of 2016, it was 100% at both hospitals. (Additional file 3 provides more details).

Second, the audits showed no change in rates of referral from baseline. Audit results for 2016 (for the 12 months after the interventions started) showed 77 and 126 patients undergoing surgery for CRC at Hospital A and B respectively. Sixteen (Hospital A) and 20 (Hospital B) patients had an abnormality on one or more of the four mismatch repair genes indicating a high risk of LS. Two patients at Hospital A and one at Hospital B had results indicating a direct referral to genetics (abnormality on MSH6), only one of whom was referred. Supplementary testing for BRAF V600E was required for 12 patients (Hospital A) and 16 patients (Hospital B). Auditors were unable to see results of some of the tests that were outsourced to other pathology services (by pathologists or treating team) early in the year, but of those that were available, seven and four patients respectively were found to be low risk. Of the two patients at Hospital A and three at Hospital B with negative BRAF V600E results indicating the need for a referral to the genetic service for LS assessment, only one of these five people was referred. All audit results are shown in Table 6.

**Table 6:** Raw data from the audits carried out by pathology and genetics on referral to the Familial Cancer Clinic

		Hospital A			Hospital B		
Quarter	Project stage	No. patients screened	No. patients requiring referral	No. patients referred	No. of patients screened	No. patients requiring referral	No. patients referred
Apr-June 14	*Baseline*	17	1	1	19	2	0
Jul-Sept 14		16	0	0	34	5	0
Oct-Dec 14		19	1	0	34	3	1
Jan-Mar 15		19	3	3	26	2	0
		**71**	**5**	**4 (80%)**	**113**	**12**	**1 (8.3%)**
Apr-June 15	*Project starts*	14	0	0	42	4	2
Jul-Sept 15		23	5	0	33	3	0
Oct-Dec 15		27	7	7	27	4	2
		**64**	**12**	**7 (58%)**	**102**	**11**	**4 (36%)**
Jan-Mar 16	*Interventions start*	23	4	1	24	2	0
Apr-June 16		19	3	3	23	3	0
Jul-Sept 16		17	2	1	45	3	1
Oct-Dec 16		18	2	1	34	3	0
		**77**	**11**	**6 (55%)**	**126**	**11**	**1 (9%)**

## Discussion

We used the TDFI approach aiming to increase the referral rate of patients with a high risk of LS into the FCC. The six steps of the TDFI approach generated high quality data: establishing there was a problem, defining barriers to change and guiding the design of interventions to address these barriers. Simplified process maps cross matched to data from the audits were shown to be highly engaging to the majority of health professionals. The maps highlighted that referrals are complex, involving many more people than the clinician filling out the actual form. Barrier data also indicated that the IHC test results were sometimes challenging to interpret and arrived in the often-fraught post-operative period, meaning referrals were often deferred.

While we were unable to demonstrate an overall increase in referrals, we can show behaviour change in earlier parts of the LS screening process. More specifically, by the end of the project, BRAF V600E testing was initiated for 100% of the patients who required it. Given that the audit and feedback intervention strategy highlighted that some clinicians believed most patients over 60 would have somatic (non-hereditary) tumours and so deemed referral to be inappropriate, this supplementary test is valuable in clarifying appropriate patients to refer for genetic testing. Further, test reports have been clarified by both pathology departments meaning results are easier to interpret; genetic specialists, counsellors, oncologists and surgeons have collaborated to introduce regular education sessions for rotating and new staff; referral forms have been added to the electronic patient record – removing the need to hunt down elusive paper referrals or write individual letters. Therefore, it may be that more time is required for these new pre-referral processes to embed into the system before the desired front end clinician behaviour change – i.e., referral of appropriate patients for genetic testing – is demonstrated. This has been seen in other implementation projects e.g. [33].

One other possible explanation for the lack of improvement in appropriate referrals may be related to our target behaviour definition: “to ensure that patients at high risk of LS are referred to the FCC.” Only the medical treating team (medical and radiation oncologists and CR surgeons) were able to make a referral yet this project promoted the view that all staff managing patients with CRC had a responsibility to ensure it happened. The target behaviour, therefore was deliberately broad to encompass the multidisciplinary team and their various roles in the referral process. For example, a pathologist might interpret “ensuring referral” to mean that their reporting should be accurate and clear, a nurse that they are alert to patient disclosures of relevant family history, or an MDT coordinator that relevant information is available for the case conference. This was useful as it meant a single version of the *IPSBQ* could be used for everyone, and it stimulated all team members to consider their individual role. However, whilst we saw pathologist test-ordering and results-reporting behaviour changes, for the medical team that made the actual referrals, there was perhaps not a clear enough statement of action. “Make a referral” seems a very straightforward behaviour, but for the medical team it was a multistep process (interpreting test results correctly, discussing with the patient, finding a referral form, faxing it, etc) reliant on other members of the department doing their part (e.g., pathologists reporting clearly). It is therefore possible that barriers to each of these more defined behaviours that make up referral could have been more targeted. Separate target behaviours for different clinical groups (e.g., relating to clearer reporting for pathologists, documenting patient disclosures of relevant family history for nurses, ensuring relevant patient family history information is provided to clinicians in MDT meetings for administrators, and using all available information to ensure all appropriate patients are referred into FCC for clinicians), may have focussed action and led to more effective intervention design [34].

Some interventions were quickly implemented (e.g., education sessions by genetic staff, clearer wording of test reports by pathologists), while others were more difficult (adding referral forms to the electronic patient management system and working out how to deal with interoperability of software systems). Michie and colleagues ([35], p. 250) recommend applying the APEASE criteria to assess potential interventions. APEASE stands for affordability, practicality, effectiveness, acceptability, side-effects/safety and equity and is a way of screening out less feasible solutions. In hindsight, use of the APEASE criteria to assess for their affordability and practicality might have allowed us to identify key but most feasible interventions.

System level factors beyond the control of the project team caused lengthy delays in implementation of some of the interventions (e.g. implementation of a new state-wide genetic database delayed introduction of new referral processes; physical relocation to a new oncology building delayed some education sessions; changes to the pathology departments’ database delayed audits). It is therefore also possible these delays have slowed the impact of interventions. An audit of referral rates of patients at high risk after a further 12 months has elapsed would be useful to determine whether such system changes had delayed results.

It is possible that some of the TDF barrier domains [21] may not have been addressed adequately with interventions. For example, ‘social/professional role and identity’ was identified as a barrier and several theoretically sound interventions were suggested to overcome this (i.e., pathologists to refer/notify oncologists of any high risk LS results; a senior oncology nurse be tasked to review all pathology results and be authorized to refer), but these were not approved by senior management. Furthermore, whilst we have been transparent about the intervention development process, the difficulties faced with attempting to work with healthcare professionals to develop theory-based interventions is a difficult task due mainly to competing pressures of service delivery. This means that the quality of the interventions may have been jeopardised, resulting in less of an impact on behaviour. This approach also makes effects difficult to unpick. Nonetheless co-design is an important factor contributing to the success of implementation activities [36, 37].

We noted early in the project that clinicians were aware of a number of different guidelines for identifying patients at high risk of LS [38, 39]. Some of these state that only patients under 50 years of age should be considered while others, such as the EviQ guidelines (see https://www.eviq.org.au/AboutUs.aspx) used at our hospitals, state “at any age” [20]. We understand this lack of consistency may have contributed to lower referral rates in the over 60 age group and would recommend that this be clarified. A consistent message is being given in the education sessions.

Change in clinical practice settings often takes a long time, especially with low incidence conditions such as Lynch syndrome. Our various attempts at changing individual health professional’s behaviour has had no impact on referral rates so far. The process is undoubtedly improved with the introduction of reflex testing but that crucial next step of appropriate review and response to these results by the treating oncologist or surgeon is not yet happening. We speculate (from anecdotal sources) that barriers to this may be that they are not actively looking for the results (since the reflex test is pathologist initiated), and if the results are reviewed, they are unfamiliar with what the results mean.

There is evidence from the US [40] that high risk LS referrals are increased in hospitals where a clinician referral is not required and genetic counsellors have direct access to genetic test results and clinical information. Again, this is worth considering implementing in other countries where the strategy is absent. Frequencies of high risk cases are low (1–2 a month) making it more feasible, although longer term solutions will need to be discussed. Genetic counsellors do not have unlimited capacity so strategies to address this should be considered.

Hospital A’s final referral rate appeared to improve more than Hospital B, however the numbers are of patients flagged at high risk are too low to draw any meaningful, statistical conclusions. We can say generally, that we observed some very engaged teams and some less interested teams at both hospitals, and we know that some teams did not have any patients identified as high risk. Others reported a reluctance to refer when patients were extremely ill, or if they died but we have no means of linking teams with individual cases.

Limitations of the study are related to the low numbers of patients affected by LS. This meant that we could not show statistically a change in the practice of referral in the time frame. Another limitation is that although we coded intuitive interventions and theory guided interventions, it is difficult to unpick mechanisms of action (or lack thereof). Process evaluation would be helpful in answering some of these questions [41].

The conventional wisdom in implementation science is to choose a high incidence problem as a target for change. Table 6 shows that the incidence of LS is low which makes new practices difficult to routinize (as they are undertaken infrequently) and change takes longer to spread to all clinicians. However, LS identification is a problem we need to solve. The health system is likely to be overwhelmed with low incidence problems as genomic testing becomes mainstream and reveals more and more low incidence hereditary cancers or syndromes. Each will require identification, appropriate care, and risk management. It is crucial for health researchers to test methods and provide health systems with generalizable solutions to address this problem so multidisciplinary teams can work together to streamline their response to this enhanced diagnostic ability.

## Conclusion

We were unable to demonstrate an overall increase in referrals, but we now have a deeper understanding of the various barriers to referral and have increased our knowledge of how to approach low frequency conditions. While referral rates did not change, the entire process from a systems perspective has been streamlined. This has enabled more accurate identification of appropriate patients and has set up an optimal context for consideration of an automatic referral system**.** The TDFI approach is currently being evaluated (in a separate project) to formally test whether the methods we used are acceptable, feasible and appropriate in a hereditary cancer setting.

## Additional files

Additional file 1: Barrier Domains and examples of matched behaviour change techniques (BCT) for use in Focus groups. (DOCX 23 kb)

Additional file 2: Oneway ANOVAs (Table A2) with Hospital as the grouping variable showed no significant difference in any of the barriers. Grouping by responsibility to refer/not responsible to refer showed a difference in the ‘skills’ domain only (*p* = 0.01). Grouping by familiarity with the guidelines for referral/those who were not familiar showed all the domains were significantly different except for ‘beliefs about capabilities’ (0.14). (DOCX 16 kb)

Additional file 3: The effect of age on appropriate supplementary testing. (DOCX 27 kb)

## Abbreviations

CRC: Colorectal cancer
FCC: Family Cancer Clinic
IHC: Immunohistochemistry
IPSBQ: Influences on Patient Safety Behaviours Questionnaire
LS: Lynch syndrome
MMR: Mismatch Repair Deficiency
NSW: New South Wales
TDFI: Theoretical Domains Framework Implementation
