# Supplementary material for: Using behaviour change and implementation science to address low referral rates in oncology
Source: BMC Health Serv Res. 2018 Nov 28;18:904. doi: 10.1186/s12913-018-3653-1 (PMC6263048; doi:10.1186/s12913-018-3653-1)
Supplement: Supplementary file 1 — Barrier Domains and examples of matched behaviour change techniques (BCT) for use in Focus groups. (DOCX 23 kb) [file 12913_2018_3653_MOESM1_ESM.docx]

**Additional File 1:** Barrier Domains and examples of matched behaviour change techniques (BCT) for use in Focus groups^[[1]](#footnote-1)^.

| **TDF domains strategy effective for** | **Technique label and definition** | **Example of strategy for patient safety** |
| --- | --- | --- |
| - **Environmental context and resources** | **BCT 13. Teach to use prompts/cues**  Teach the person to identify environmental prompts which can be used to remind them to perform the behaviour. This could include times of day, particular contexts or elements of contexts which prompt them to perform the target behaviour. | Screensavers/posters with key information to remind staff about using pH to check position; computerised prompts for ensuring blood levels for specific patients are checked on time |
| - **Environmental context and resources** | **BCT 23. Time management**  This includes any technique designed to help a person make time for the behaviour (e.g., how to fit it into a daily or weekly schedule). These techniques are not directed towards performance of target behaviour but rather seek to facilitate it by freeing up times when it could be performed. This technique may or may not be mentioned by name. | Ensure there is space blocked out within the specific time window for a blood test for patients who have received gentamicin |
| - **Social influences** | **BCT 3. Provide information about others’ approval**  Involves information about what other people think about the reader’s or target person’s behaviour. It clarifies whether others will approve or disapprove of what the person is doing or will do. | Use posters or screensavers with pictures of senior members of staff advocating the behaviour |
| - **Social influences** | **BCT 6. Provide general encouragement**  Involves praising or rewarding the person for effort or performance without making this contingent on specific behavioural performance; or “motivating” the person in an unspecified manner. This will include attempts to enhance self-efficacy through argument or persuasion (e.g., telling someone the will be able to perform a behaviour). | Provide praise for attempting to use pH as first line method for checking nasogastric tube position |
| - **Social influences** | **BCT 16. Provide opportunities for social comparison**  This will most commonly be seen in the case of group practice (e.g., group classes) but could also be employed using detailed case studies in text or video or by pairing people as supports. It provides a setting in which processes such as social comparison could occur. | Obtain results of another ward for use of pH as first line method to check NG tube position to compare and contrast efforts, progress |
| - **Social influences** | **BCT 17. Plan social support/ social change**  Involves prompting the person to think about how others’ could change their behaviour to offer him/her help and/or (instrumental) social support. This will also include provision of such support during the interventions e.g., setting up a “buddy” system or other forms of support. | Team up junior doctors with experienced nurse ‘buddys’ to remind them to check blood levels of gentamicin patients within specified time window |
| - **Social influences** | **BCT 18. Prompt identification as role model/ position advocate**  Involves focusing on how the person may be an example to others and affect their behaviour (e.g. being a good example to children). Also includes providing opportunities for participants to persuade others of the importance of adopting/ changing the behaviour (e.g. giving a talk or writing a persuasive leaflet). | Inform nursing staff that junior doctors look up to them, so they need to set the right example so good practice is sustained |
| - **Skills** | **BCT 7. Set graded tasks**  Set the person easy-to-perform tasks, making them increasingly difficult until target behaviour is performed. | Practice obtaining aspirate on manikin before moving to real person |
| - **Skills** | **BCT 8. Model/ Demonstrate the behaviour**  Involves showing the person how to correctly perform a behaviour e.g., face-to-face as in a group class or using video. | Show someone how to manoeuvre a patient in order to increase chances of obtaining aspirate from stomach |
| - **Skills** | **BCT 14. Prompt practice**  Prompt the person to rehearse and repeat the behaviour or preparatory behaviours numerous times. Note this will also include parts of the behaviour e.g., refusal skills in relation to quitting smoking. This could be described as “building habits or routines” but is still practice so long as the person is prompted to try the behaviour (or parts of it) during the intervention. | Provide training with manikins for obtaining aspirate and moving patients in order to increase chances; simulation centre training for teams working with a deteriorating patient to practice using skills and developing situational awareness |
| - **Action planning** | **BCT 4. Prompt intention formation**  Involves encouraging the person to set a general goal or make a behavioural resolution e.g., “I will take more exercise next week” would count as a prompt to intention formation. This is directed towards encouraging people to decide to change. | Commit to writing prescriptions in capital letters over the next week |
| - **Action planning** | **BCT 12. Provide contingent rewards**  This can include praise and encouragement as well as material rewards but the reward/ incentive must be explicitly linked to the achievement of specified goals i.e. the person receives the reward if they perform the specified behaviour (or preparatory behaviour) but not if they do not perform the behaviour. | Budget for additional training in patient safety area of choice once target met; or provide lunch for staff once target met |
| - **Action planning** | **BCT 13. Teach to use prompts/cues**  Teach the person to identify environmental prompts which can be used to remind them to perform the behaviour. This could include times of day, particular contexts or elements of contexts which prompt them to perform the target behaviour. | Screensavers/posters with key information to remind staff about using pH to check position; computerised prompts for ensuring blood levels for specific patients are checked on time |
| - **Action planning** | **BCT 20. Relapse prevention**  Following an initial change help the person identify situations that increase the likelihood of returning to a risk behaviour or failing to perform a new health behaviour – and help them plan how to avoid or manage the situation so that new behavioural routines are maintained | Anticipate a situation that might prevent you from using pH paper (e.g., can’t find pH so easier to send for x-ray) and plan a way to overcome this (e.g., ensure enough stock for ward) |
| - **Motivation and goals** | **BCT 1. Provide general information on behaviour-health link**  Information about the relationship between the behaviour and health – including susceptibility or factual risk and/or mortality information OR. health education material relevant to the behaviour. | Provide guidelines for practice which are supported by evidence |
| - **Motivation and goals** | **BCT 3. Provide information about others’ approval**  Involves information about what other people think about the reader’s or target person’s behaviour. It clarifies whether others will approve or disapprove of what the person is doing or will do. | Use posters or screensavers with pictures of senior members of staff advocating the behaviour |
| - **Motivation and goals** | **BCT 2. Provide information on consequences**  Involves providing information focusing on what will happen if the person performs the behaviour including the benefits and costs of action or inaction. | Provide information about number of hospital acquired infections caused by not washing hands |
| - **Motivation and goals** | **BCT 6. Provide general encouragement**  Involves praising or rewarding the person for effort or performance without making this contingent on specific behavioural performance; or “motivating” the person in an unspecified manner. This will include attempts to enhance self-efficacy through argument or persuasion (e.g., telling someone the will be able to perform a behaviour). | Provide praise for attempting to use pH as first line method for checking nasogastric tube position |
| - **Motivation and goals** | **BCT 7. Set graded tasks**  Set the person easy-to-perform tasks, making them increasingly difficult until target behaviour is performed. | Practice obtaining aspirate on manikin before moving to real person |
| - **Motivation and goals** | **BCT 8. Model/ Demonstrate the behaviour**  Involves showing the person how to correctly perform a behaviour e.g., face-to-face as in a group class or using video. | Show someone how to manoeuvre a patient in order to increase chances of obtaining aspirate from stomach |
| - **Motivation and goals** | **BCT 10. Prompt review of behavioural goals**  Involves reconsideration of previously set goals/ intentions. In most cases this will follow previous goal setting and an attempt to act on those goals. | Assess progress towards goals for taking blood levels of gentamicin patients within a specified time period, and amend as appropriate (to make them easier or harder depending progress) |
| - **Motivation and goals** | **BCT 5. Prompt barrier Identification**  Think about potential barriers and plan ways of overcoming them. Barriers may include competing goals in specified situations. This may be described as “problem solving” and if it is problem solving in relation performance of the behaviour then it is an instance of this technique. | Identify barriers to titrating an injectable medicine to patient’s individual needs, and work through ways to overcome them |
| - **Motivation and goals** | **BCT 6. Provide general encouragement**  Involves praising or rewarding the person for effort or performance without making this contingent on specific behavioural performance; or “motivating” the person in an unspecified manner. This will include attempts to enhance self-efficacy through argument or persuasion (e.g., telling someone the will be able to perform a behaviour). | Provide praise for attempting to use pH as first line method for checking nasogastric tube position |
| - **Motivation and goals** | **BCT 9. Prompt specific goal setting**  Involves detailed planning of what the person will do including, at least, a very specific definition of the behaviour e.g., frequency (such as how many times a day/week), intensity (e.g., sped) or duration (e.g., for how long for). In addition, at least one of the following contexts i.e., where, when, how or with whom must be specified. This could include identification of sub-goals or preparatory behaviours and/or specific contexts in which the behaviour will be performed. | Encourage setting goal to ensure that I use pH as the first line to check NG tube position on the next three NG tube patients that I see |
| - **Motivation and goals** | **BCT 20. Relapse prevention**  Following an initial change help the person identify situations that increase the likelihood of returning to a risk behaviour or failing to perform a new health behaviour – and help them plan how to avoid or manage the situation so that new behavioural routines are maintained | Anticipate a situation that might prevent you from using pH paper (e.g., can’t find pH so easier to send for x-ray) and plan a way to overcome this (e.g., ensure enough stock for ward) |
| - **Motivation and goals** | **BCT 7. Set graded tasks**  Set the person easy-to-perform tasks, making them increasingly difficult until target behaviour is performed. | Practice obtaining aspirate on manikin before moving to real person |
| - **Motivation and goals** | **BCT 10. Prompt review of behavioural goals**  Involves reconsideration of previously set goals/ intentions. In most cases this will follow previous goal setting and an attempt to act on those goals. | Assess progress towards goals for taking blood levels of gentamicin patients within a specified time period, and amend as appropriate (to make them easier or harder depending progress) |
| - **Motivation and goals** | **BCT 12. Provide contingent rewards**  This can include praise and encouragement as well as material rewards but the reward/ incentive must be explicitly linked to the achievement of specified goals i.e. the person receives the reward if they perform the specified behaviour (or preparatory behaviour) but not if they do not perform the behaviour. | Budget for additional training in patient safety area of choice once target met; or provide lunch for staff once target met |
| - **Motivation and goals** | **BCT 17. Plan social support/ social change**  Involves prompting the person to think about how others’ could change their behaviour to offer him/her help and/or (instrumental) social support. This will also include provision of such support during the interventions e.g., setting up a “buddy” system or other forms of support. | Team up junior doctors with experienced nurse ‘buddys’ to remind them to check blood levels of gentamicin patients within specified time window |

1. [Adapted from Taylor, N., et al, Achieving Behaviour Change for Patient Safety Toolkit. Yorkshire & Humber Academic Health Services Network, Improvement Academy http://www.improvementacademy.org/resources/abc-for-patient-safety-workshop-and-toolkit/] [↑](#footnote-ref-1)
