# Supplementary material for: Using behaviour change and implementation science to address low referral rates in oncology
Source: BMC Health Serv Res. 2018 Nov 28;18:904. doi: 10.1186/s12913-018-3653-1 (PMC6263048; doi:10.1186/s12913-018-3653-1)
Supplement: Supplementary file 2 — Oneway ANOVAs (Table A2) with Hospital as the grouping variable showed no significant difference in any of the barriers. Grouping by responsibility to refer/not responsible to refer showed a difference in the ‘skills’ domain only (p = 0.01). Grouping by familiarity with the guidelines for referral/those who were not familiar showed all the domains were significantly different except for ‘beliefs about capabilities’ (0.14). (DOCX 16 kb) [file 12913_2018_3653_MOESM2_ESM.docx]

# Additional File 2:

Oneway ANOVAs (Table A2) with Hospital as the grouping variable showed no significant difference in any of the barriers. Grouping by responsibility to refer/not responsible to refer showed a difference in the ‘skills’ domain only (p = 0.01). Grouping by familiarity with the guidelines for referral/those who were not familiar showed all the domains were significantly different except for ‘beliefs about capabilities’ (0.14).

**Table A2: ANOVAs of mean barriers grouped by hospital at which respondent works, familiarity with Lynch syndrome referral guidelines, and whether respondent has responsibility to refer high risk patients.**

| Barrier domain | Hospital | | |  | Familiarity with guidelines | | |  | Responsibility to refer | | |
| --- | --- | --- | --- | --- | --- | --- | --- | --- | --- | --- | --- |
|  | **F (df)** | **p value** | **Higher barrier for** |  | **F** | **p value** | **Higher barrier for** |  | **F** | **p value** | **Higher barrier for** |
| Beliefs about capabilities | 1.18 (1,34) | 0.29 | NA |  | 2.29 (1,34) | 0.14 | NA |  | 0.00 (1,34) | 1.00 | NA |
| Knowledge | 0.14 (1,34) | 0.91 | NA |  | 14.66 (1,34) | **0.00** | Not familiar |  | 0.46 (1,34) | 0.50 | NA |
| Professional identity | 0.23 (1,34) | 0.64 | NA |  | 15.16 (1,34) | **0.00** | Not familiar |  | 2.67 (1,34) | 0.11 | NA |
| Beliefs about consequences | 2.59 (1,34) | 0.12 | NA |  | 6.58 (1,34) | **0.02** | Not familiar |  | 1.12 (1,34) | 0.30 | NA |
| Motivation and goals | 1.08 (1,34) | 0.31 | NA |  | 5.71 (1,34) | **0.02** | Not familiar |  | 1.05 (1,34) | 0.31 | NA |
| Memory, attention and decision-making | 0.19 (1,34) | 0.67 | NA |  | 27.79 (1,34) | **0.00** | Not familiar |  | 0.49 (1,34) | 0.49 | NA |
| Environmental context and resources | 0.11 (1,34) | 0.75 | NA |  | 5.52 (1,34) | **0.03** | Not familiar |  | 0.25 (1,34) | 0.62 | NA |
| Emotion | 0.37 (1,34) | 0.55 | NA |  | 8.35 (1,34) | **0.01** | Not familiar |  | 1.05 (1,34) | 0.31 | NA |
| Action planning | 1.77 (1,34) | 0.19 | NA |  | 5.90 (1,34) | **0.02** | Not familiar |  | 3.39 (1,34) | 0.08 | NA |
| Skills | 0.01 (1,34) | 0.94 | NA |  | 5.12 (1,34) | **0.03** | Not familiar |  | 6.79 (1,34) | **0.01** | Not respons-ible |
