# Supplementary material for: Using behaviour change and implementation science to address low referral rates in oncology
Source: BMC Health Serv Res. 2018 Nov 28;18:904. doi: 10.1186/s12913-018-3653-1 (PMC6263048; doi:10.1186/s12913-018-3653-1)
Supplement: Supplementary file 3 — The effect of age on appropriate supplementary testing. (DOCX 27 kb) [file 12913_2018_3653_MOESM3_ESM.docx]

**Additional File 3**

**The effect of age on appropriate supplementary testing.**

Analysis of the audit results revealed a clear age difference between those referred and those not referred. Younger patients, under 50 years old were consistently being identified as high risk, usually before their surgery, and so received referrals. Older patients, especially those over 70 years were not, even though the Immunohistochemistry (IHC) test results indicated abnormalities. Older guidelines (e.g., the Amsterdam and Bethesda protocols ^(1)^) familiar to some of the clinicians had previously had only considered patients under the age of 50 in their criteria. This had more recently been revised in the EviQ guidelines used in Australia^(2)^ to include the wording “abnormalities at any age.”

The audit results were re-analysed in late 2016 (33 months data including the 12 month retrospective audit) to examine more closely patterns around which patients were being missed. The most common abnormalities seen over all were on both the MLH1 and PMS2 genes. These patients required supplementary testing (BRAF V600E) to screen out patients unlikely to be carrying LS. We found a significant number of patients with abnormal MLH1 and PMS2 abnormalities missing this supplementary test who were over 50 years of age. We used an audit and feedback intervention to report this back to each hospital’s multidisciplinary teams and confirm that latest guidelines recommended supplementary testing at any age.

As pathology departments became able to provide this supplementary testing in-house, individual pathologists started to offer this test reflexively (i.e., without a formal request from the treating doctors). By the end of the project, patients requiring supplementary testing were consistently receiving it. While still an *ad hoc* process, pathology departments are working towards formalising this.

1. Vasen H, Moslein G, Alonso A, Bernstein I, Bertario L, Blanco I, et al. Guidelines for the clinical management of Lynch syndrome (hereditary non-polyposis cancer). Journal of Medical Genetics. 2007;44:353-62.

2. EviQ. Genetic testing for hereditary mutations in the mismatch repair genes (MMR-genes) 2016 [Available from: https://www.eviq.org.au/Protocol/tabid/66/id/619/defid/11004/Default.aspx?popup=1.
